# Cell Heterogeneity and Paracrine Interactions in Human Islet Function: A Perspective Focused in β-Cell Regeneration Strategies

**DOI:** 10.3389/fendo.2020.619150

**Published:** 2021-02-03

**Authors:** Eva Bru-Tari, Daniel Oropeza, Pedro L. Herrera

**Affiliations:** Department of Genetic Medicine and Development, Faculty of Medicine, University of Geneva, Geneva, Switzerland

**Keywords:** human islet, beta-cell regeneration, paracrine signaling, cell heterogeneity, glucose homeostasis

## Abstract

The β-cell regeneration field has shown a strong knowledge boost in the last 10 years. Pluripotent stem cell differentiation and direct reprogramming from other adult cell types are becoming more tangible long-term diabetes therapies. Newly generated β-like-cells consistently show hallmarks of native β-cells and can restore normoglycemia in diabetic mice in virtually all recent studies. Nonetheless, these cells still show important compromises in insulin secretion, cell metabolism, electrical activity, and overall survival, perhaps due to a lack of signal integration from other islet cells. Mounting data suggest that diabetes is not only a β-cell disease, as the other islet cell types also contribute to its physiopathology. Here, we present an update on the most recent studies of islet cell heterogeneity and paracrine interactions in the context of restoring an integrated islet function to improve β-cell replacement therapies.

## Introduction

The islets of Langerhans are complex micro-organs composed of different endocrine cell types whose principal function is the maintenance of glucose homeostasis and feeding behavior through coordinated hormone secretion and paracrine interactions. Different studies have estimated the human islet to be comprised mainly by insulin-secreting β-cells in the range of 52–75% (1–5). Following in number, glucagon-secreting α-cells and somatostatin-secreting δ-cells comprise some 40 and 10% of the islet. Pancreatic polypeptide (PP)-secreting γ-cells and ghrelin-secreting ϵ-cells are the minor cell types comprising about 5% and less than 1% of the islet, respectively. Islets cells are characterized by an exquisite secretory capacity and cell mass modulation that efficiently adapts to diverse metabolic stresses or pathologies like pregnancy and obesity. Defects in this adaptive capacity are at the core of certain impairments in nutrient metabolism and diabetes development (6–8).

The different islet cell types are arranged in an intricate network that facilitates cell proximity and direct contacts that fine-tune hormone secretion to robustly control glucose homeostasis. In human islets, there is a predominance of heterologous contacts between β- and α-cells, suggesting their direct interaction is crucial for glycaemia management (9). Indeed, paracrine α-cell signaling establishes a set point for insulin secretion and glycemia throughout different animal species (10). Dysregulation of islet paracrine interactions and non-β-cell function contribute substantially to diabetes symptomatology (11–17). This could be one of the reasons why conventional diabetes therapies relying solely on exogenous insulin do not maintain stable normoglycemia. It is becoming apparent that diabetes is a disease concerning the whole islet, yet regenerative approaches have for the most part focused mainly on restoring a functional β-cell mass and basal insulin secretion with little regard on achieving a balanced islet secretory output. Here, we present an update on recent studies highlighting the importance of islet paracrine interactions and cell heterogeneity for a highly malleable human islet function that achieves optimal glucose homeostasis and withstands the range of stresses present in the ever-changing physiologic and cellular environment.

## Diabetes Affects All Islet Endocrine Cell Types

Mounting data shows that the complex islet cytoarchitecture, gene expression, and function of non-β-cells are also significantly compromised throughout diabetes progression. Both T1D and T2D patients present hyperglucagonemia in postprandial conditions or upon oral glucose challenge, that exacerbates hyperglycemia (11–15). The reasons are not fully understood, yet i) lack of intra-islet insulin, ii) dysfunctional α-cell glucose sensing, or iii) increases in the functional α-cell mass may be underlying mechanisms. A convoluted combination of these defects may also be possible, as T2D patients present hyperglucagonemia even in the fasting state (16, 17) while T1D patients present a defective α-cell response to hypoglycemia (18).

In T1D, α-cell mass is maintained in the early stages of the disease (19) while it clearly decreases in advanced stages (20). For T2D, most published studies do not specify the stage of the disease, and report conflicting results depending on the analytical method, with a higher α- to β-cell ratio in long-standing T2D pancreas (9, 21, 22) or a decrease of the total glucagon+ area in all regions of the pancreas (23), but no differences in α-cell mass (22). This suggests that α-cell defects may not be due to an increased cell mass. In concordance, recent studies with islets from T2D donors show no inhibition of glucagon secretion *in vitro* at high glucose concentrations (24). Moreover, transplantation of islets from T2D donors into a novel Glucagon knockout-NSG mouse model showed increased glucagon secretion during fasting and upon insulin-induced hypoglycemia, suggesting that hyperglucagonemia in T2D is caused by local islet defects that are not resolved when transplanted into a non-diabetic environment (25). Nonetheless, the effect of induced hyperglycemia was not tested in this study. In addition, single cell RNA sequencing (scRNA-seq) of α- and δ-cells from T2D donors showed a downregulation of energy metabolism and protein synthesis genes (26, 27). In contrast, islets from T1D donors show decreased glucagon secretion at low glucose concentrations *in vitro* (28), underlying the high risk of severe hypoglycemia after insulin administration in T1D patients (18, 28). α-cells from T1D donors also show differential gene expression, including in electrical activity and exocytosis, as well as master regulators of α-cell identity, *ARX* and *MAFB* (28).

Only few studies tested the effect of diabetes on the minor islet cell types, yet it is likely they are also significantly affected. ϵ-cells show a reduction in their number that could be linked to lower plasma ghrelin levels in T2D (29). Yet, this is unlikely since most ghrelin-secreting cells are extrapancreatic (gastric fundus). In δ-cells, islets from T2D donors showed blunted *in vitro* somatostatin secretion in response to glucose while some donors show hypersecretion at low glucose (30). While there are no reports on δ-cells from T1D donors, recent findings in diabetic mice indicate that increased somatostatin signaling may be reducing counter-regulatory glucagon secretion during insulin-induced hypoglycemia (31). Finally, T2D patients present high plasma levels of PP after an oral glucose challenge (32). As PP inhibits somatostatin secretion in human islets (33), is possible that increased levels of PP contribute to diabetic hyperglucagonemia by decreasing the somatostatin inhibitory effect on α-cells.

Collectively, these studies suggest that diabetes eventually becomes an islet disease affecting all islet cells or that the degree of initial non-β-cell dysfunction is a contributing factor accelerating the progression or the severity of diabetes. Whether non-β-cell defects are intrinsic or solely the result of the decrease in β-cells and local insulin, is still the focus of intense research. Little is known about the role of non-β-cell function in glucose intolerance, prediabetes or the initial stages of diabetes. δ-Cell electrical oscillatory activity in response to glucose stimulation is impaired in insulin resistant mice treated with high fat diet (34) and non-human primates show decreased proportion of δ-cells per islet that progresses with mounting hyperglycemia, possibly caused by δ-cell apoptosis (35). Alterations in δ-cell secretory function during the progression of type 2 diabetes may exacerbate β-cell exhaustion due to a lack of inhibitory signals exerted by somatostatin or could be an adaptation to the higher insulin demand during prediabetes. In the case of α-cells, insulin resistant and glucose intolerant mice under high fat diet present α-cell hypertrophy and lack of suppression of glucagon release upon intraperitoneal glucose injection (36). Studies with obese subjects also observed hyperglucagonemia upon postprandial conditions (37) and in non-human primates α-cell mass tends to increase with the duration and severity of obesity (38). It has been postulated that α-cell insulin resistance (39), intrinsic defects in α-cell glucose sensing or a reduced somatostatin signaling may lead to α-cell functional alterations at this stage (40). Nonetheless, these observations also highlight that defects in non-β-cells may appear in the eventual progression to T2D.

Analysis of non-β-cell numbers and circulating levels of their corresponding hormones in prediabetes or the initial stages of diabetes is needed to understand when these defects start and their contribution to diabetes before β-cell function is impaired. Likewise, whether defective α-cell function in diabetes is completely restored by the regeneration of a functional β-cell mass is still not clear. T1D recipients of islet transplants showed an absence or only partial restoration of glucagon secretion upon insulin-induced hypoglycemia (41–43). The combination of mouse diabetic models showing dysregulated glucagon secretion (44, 45) that allow the measurement of human plasmatic glucagon (25) and transplantation of purified human α-cells alone or in combination with other islets cells will be needed to address this issue.

## Human Islet Architecture Favors Heterologous Contacts Between Endocrine Cells: Implications in The Counter-Regulatory Islet Response to Hypoglycemia

**Figure 1:**
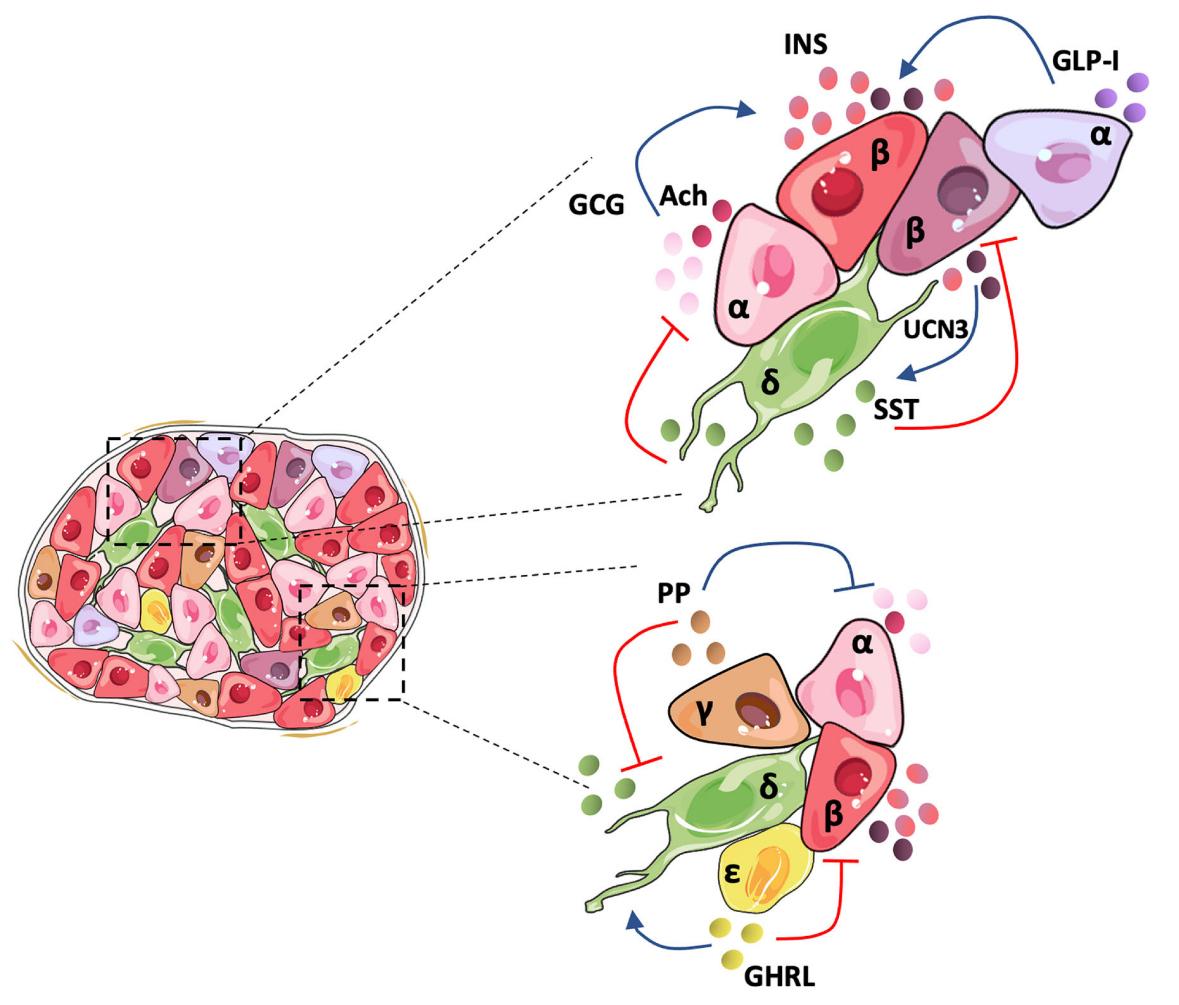
Human islet architecture favors heterologous contacts and holds a tightly regulated cellular inter-communication network. In the human islet, β-and non-β-cells present frequent contacts favoring paracrine signaling between the cells. Human α-cells secrete mainly glucagon (GCG), acetylcholine (Ach), and GLP-I, which activates insulin secretion by β-cells. It has been postulated that an α-cell subpopulation holds the intra-islet secretion of GLP-I. Human pancreatic β-cells comprise a heterogeneous population and may hold differences in the amount of insulin (INS) secreted depending on the subpopulation. UCN3 is secreted along with INS and activates somatostatin (SST) release by δ-cells. SST inactivates GLC and INS secretion, closing the loop of paracrine signaling between the main islet cell types. Although less studied, the minor islet cell types also contribute to the regulation of the islet hormone secretion. Pancreatic polypeptide (PP) secreted by γ-cells suppresses both GCG and SST release, and ghrelin (GHRL), produced by ϵ-cells, seems to activate SST and inhibit INS release.

In 1982, pioneering studies described a poor responsiveness of isolated single rat β-cells to glucose, an effect that was linked to the lack of α-cell contacts and glucagon, revealing the crucial role of the islet architecture in the optimal functional cooperation between islet cells (46). Since then, most studies have focused on the core-mantle arrangement of rodent islets that clusters β-cells in the center surrounded by peripheral non-β-cells (4). This favors homologous β-to-β cell contacts shown to be critical in mice for regulating *in vivo* insulin secretory dynamics and glucose homeostasis through gap-junction coupling (47), which drives β-cell synchronization in terms of electrical activity and intracellular calcium concentration. However, it is broadly accepted that human islet cell types are distributed more randomly (3, 4). Recent studies show that the human islet involves a more intricate structure that depends on islet size. Small human islets (40-60 µm in diameter), which are more frequent during childhood, display the core-mantle structure of rodent islets, while large islets are formed by multiple subunits of β-cell clusters surrounded by non-β cells, containing a lower proportion of β-cells than the smaller ones (48). A similar trend is observed between juvenile and aged mice. This unique arrangement presents a higher rate of heterologous contacts, while maintaining homologous contacts between β-cells (9). In the case of humans, β-cells seem to be less synchronized than in mice in response to stimulatory glucose concentrations (4), possibly due to their organization within the islet, which can prime them to have a weaker β-cell electrical coupling. Indeed, synchronous intracellular calcium oscillations in response to stimulatory glucose concentrations have been recorded only in β-cells within the same islet region (49). The higher rate of heterologous contacts within the human islet suggests that counter-regulatory paracrine interactions might play a more important role in human than in mouse islets for the fine tuning of insulin secretion and glycemia maintenance. **Figure 1** summarizes paracrine interactions between human islet cell types.

In humans, α-cell signaling potentiates insulin secretion throughout a wide range of glucose concentrations (50), establishes the glycemic set point for insulin secretion (10) and enhances insulin secretion when β-cells are in contact with α-cells (51). This is classically known to be mediated by glucagon signaling (10), which activates human β-cell G protein-coupled receptors (GPCR) of class B, including glucagon receptor (GCGR), and glucagon-like peptide 1 receptor (GLP-1R), promoting insulin secretion by an increase in cyclic AMP and the recruitment of insulin granules (50). However, GLP-1 is also secreted by human α-cells (52–54) and necessary for insulin secretion in human islets, as GLP-1R antagonism blunted glucose stimulated insulin secretion (GSIS) *in vitro* (54). Moreover, GLP-1 can elicit synchronous intracellular calcium oscillations in human whole islets (55), suggesting an important role of this hormone and the location of α-cells within the islet to obtain pulsatile and synchronized insulin secretion. Human α-cells also amplify GSIS through the parasympathetic neurotransmitter acetylcholine, which is co-secreted with glucagon (56, 57) and acts through the activation of the muscarinic receptor M3 in β-cells (57). It has been postulated that this mechanism aids in maintaining β-cell responsiveness to the subsequent rise in glucose produced by glucagon action (56). Subsequently, excessive insulin release and hypoglycemia are avoided by a paracrine negative feedback loop between β- and δ-cells (58), mediated by urocortin-3 (UCN3), which is released by β-cells along with insulin. UCN3 activates type 2 corticotropin-releasing hormone receptors specifically borne by δ-cells within the human islet and stimulates somatostatin secretion (58), which directly inhibits insulin secretion through somatostatin receptor 2 (SSTR2) activation (59). Recent data shows that human δ-cells have long filopodia containing secretory granules that allow for direct contact with multiple β- and α-cells (34), and suggests that human β- and δ-cells are coupled by gap junctions (60), as somatostatin secretion follows the same pulsatile and coordinated response of insulin secretion in isolated islets (61).

Recent data is also starting to shed light on how paracrine interactions control α- and δ-cell secretory outputs. The activation of somatostatin secretion by β-cells, directly inhibits glucagon secretion through SSTR2 in α-cells (31) and *in vitro* chemical inhibition of insulin or somatostatin signaling in whole human islets induces glucagon secretion at non-stimulatory glucose concentrations (24). Isolated human α-cells also activate glucagon secretion at non-stimulatory glucose concentrations, which was corrected by reaggregation with purified β-cells but not by incubation with β-cell secreted factors (62). Lastly, ghrelin has been recently shown to suppress insulin secretion in human islets (29) and potentiate somatostatin release (63), thus suggesting a novel role for ϵ-cells in the control of hypoglycemia. Conversely, γ-cells seem to enhance human insulin secretion through an inhibition in somatostatin secretion caused by the PP activation of NPYR4 receptor in δ-cells (33). PP also activates the PPYR1 receptor (which is present in human α-cells) in mouse α-cells and inhibits glucagon secretion (64).

Knowledge about how human islet structure and the integrated input of paracrine signaling control synchronization of β-cells and islet hormone secretion is scarce and fragmented in comparison with the mouse islet. While mouse islets present tightly synchronized β-cell function and less heterologous cell contacts, non-β-cells might play a more important role in human islets. New techniques employing high yields of purified primary human islet cells will be necessary to study the contribution of each cell type to integrated islet function. Systematic and high-resolution secretory profiling under various metabolic stresses, using isolated as well as reaggregated human islet cells in different combinations, will help dissect the bewildering interplay of interactions that coordinate optimal islet secretion. A recent study employing reaggregated human islets and a microfluidic system that measures secretion concurrently with intracellular signaling dynamics highlights the importance of developing new tools to study human islet cells, as it revealed G_i_ GPCR signaling decreases insulin and glucagon secretion while G_q_ GPCRs stimulate glucagon secretion but have dual effects on insulin secretion (65).

## Human Islets are Comprised of Heterogeneous Cell Populations: Relevance in β-Cell Function and Stress Adaptation

A higher level of complexity stems from recently identified β- and α-cell subpopulations based on physiological (66–68), transcriptomic (27, 67, 69–72), and proteomic differences (73–75). Upon stimulatory conditions, human β-cells located in discrete islet regions synchronize calcium flux and electrophysiology (4, 49, 76). Shedding light on whether this is due to their location in the islet, their specific β-β physical interactions or to intrinsic features, employing functional cell mapping with optogenetics, “hub” β-cells (10% of the human islet) were identified as first-responders that engage other β-cells into insulin secretion (66). These cells are considered immature based on low *Pdx1* and *Nkx6.1* expression and low insulin content (66). In mouse islets, “hub” β-cell function is not affected by the inhibition of glucagon signaling or by their location within the islet (66). However, these features have not been explored in human cells, neither whether “hub” cells have a higher number of contacts with α-cells or δ-cells, which could hint at which cell type has a bigger functional influence on this β-cell subpopulation.

Interestingly, “hub” β-cells are more susceptible to glucolipotoxicity, resulting in reduced numbers and high glucokinase protein levels (66). The genes involved in responses to different metabolic insults [including unfolded protein response (UPR), endoplasmic reticulum (ER) stress, and oxidative stress] efficiently cluster β-cells into subpopulations (69–71). This is one of the most relevant features across different scRNA-seq analyses of β-cell heterogeneity, although there is no consensus on the transcriptomic identity of β-cell subpopulations (27, 69–71). In one instance, three β-cell sub clusters where observed matching low UPR with low insulin expression, low UPR with high insulin, and high UPR with low insulin (69). These groups might be transiently moving between a state high insulin production and secretion to fulfill the requirements that maintain normoglycemia, and a state of UPR-mediated recovery from ER stress due to high insulin production, taking the role of hub cells that orchestrate secretion from neighboring cells (69). Importantly, the heterogeneity in UPR responses may have a significant impact on the survival of β-cells to metabolic insults as chronic UPR activation is present in islets from T2D donors and those at risk to suffer the disease (77). However, other studies have not observed differences in UPR-related genes or correlation of any of these β-cell subpopulations with obesity or T2D (27, 72).

In the case of human α-cells, several scRNA-seq analyses identified human α-cell subpopulations with a proliferative profile (27, 69, 72), which have also been reported in pancreatic sections of adolescents (78) and can be correlated with a lower expression of UPR genes (69). At the functional level, GLP-1 secretion has been linked to specific α-cell subgroups that are more prevalent in T2D, indicating a possible α-cell adaptation to higher insulin demand (68). At the structural level, α-cells can be divided into subsets containing different ranges of glucagon granules (75), which suggest distinct secretory properties, yet no heterogeneity in glucagon secretion has been reported (68). However, studies in mice have shown that α-cell subsets vary in calcium flux and membrane capacitance upon stimulatory conditions (45, 79), which may correlate with granule density.

Studies that connect transcriptionally distinct subpopulations with β- and α-cell function are scarce due to technical limitations. Initial reports linked the lack of cell surface markers CD9 and ST8SIA1 with a β-cell subpopulation showing decreased insulin secretion (73). More recently, a study combining scRNA-seq with patch-clamp electrophysiological measurements of vesicle exocytosis and ion-channel activity, found improved excitability properties in a subpopulation of low-expressing *RBP4* β-cells in non-diabetic donors, as well as α-cell electrophysiological heterogeneity correlated with differential expression of ER stress markers in non-diabetic and T2D samples (67). Novel methods to inactivate specific subpopulations will unravel the role of heterogeneity in islet function.

## Concluding Remarks: Targeting Islet Paracrine Interactions and Heterogeneity for Optimal and Robust Islet Function in β-Cell Regeneration Strategies

Available data suggests that diabetes affects all islet cell types. Islet paracrine interactions and heterogeneity are key features that allow adaptation to a wide spectrum of physiological challenges. Consequently, β-cell regeneration strategies must consider these factors to restore optimal islet secretory capacity. Indeed, islet transplantation, which would partially replenish a functional islet mass, restores circulating insulin to comparable levels of healthy individuals (80, 81), although efficient glucagon secretion upon hypoglycemia is only partially restored (41, 42). It is unclear if transplanting only β-cells to diabetic patients would give similar results, but analogous experiments could be performed in diabetic mice using different combinations of purified human islet populations. *De novo* generation of surrogate or replacement β-cells from stem cells (SC-β), or other cell sources, has focused in achieving insulin production and secretion comparable to native β-cells under stable conditions. Recent protocols yield SC-β cells that reverse hyperglycemia in mice for up to 45 days, with detectable human C-peptide within 3–14 days after transplantation (82–86). Despite this amazing progress, SC-β cells do not achieve the biphasic insulin release nor the magnitude of insulin secretion of cadaveric islets *in vitro*, possibly because of a disconnection in glucose sensing (87). Moreover, long-term analysis or the whole range of metabolic stresses including pregnancy, obesity, pathogenic infection, or extreme fasting [where β-cells undergo major modifications which must be quickly reversed after refeeding (88)] have not been explored. Besides, the impact of cell heterogeneity in SC-β strategies remains elusive, with only one report indicating that β-cell subpopulations were not detected after SC-β transplantation in mice (89). Although there are not such studies in human, heterogeneity is crucial in mice for β-cell adaptation to pathological stressors like obesogenic diets (90).

The signals coming from a diverse non-β-cell population might be pivotal to maintain robust insulin content and secretion throughout all these conditions. As described above, while mouse islets seem to rely on homologous β-cell contacts to achieve a synchronized function, in humans, non-β-cells may play a fundamental role as heterologous contacts are more prevalent and GLP-I signaling elicits a coordinated β-cell activation (55). Moreover, functional non-β-cells could also be required for the maturation, as glucagon receptor KO mice show lower expression of Pdx1, Glut2, and MafA in β-cells (91). Additionally, decreased insulin content occurs in glucagon-GFP knock-in mice that lack proglucagon derived peptides (92) and human insulin promoter activity is stimulated by GLP-1 (93). The implications of the cellular architecture and cell diversity in the generation of functional SC-β-cells has been studied recently by the generation of human islet-like organoids (94), containing some 60% of cells co-expressing insulin and other key β-cell markers, along with glucagon, somatostatin, and PP-positive cells (94). This improved functional maturation of SC-β-cells in terms of GSIS and, after the transplantation in mice, allowed for controlled insulin secretion upon a cycle of feeding, fasting, and refeeding (94). Although controls with only SC-β cells is needed to prove if this tuned insulin secretion is driven by adjacent non-β cells, the presence of UCN3 at protein level in β-cells, suggests that paracrine signaling may be restored (94).

Overall, non-β-cell paracrine signaling is key for optimal islet hormone secretion and any disruption to this balanced cell system may exacerbate diabetes symptomatology and compromise β-cell function. In diabetes, non-β-cells present defects that may be not rectified by the sole reestablishment of insulin signaling *via* β-cell regeneration approaches. This data supports the idea that non-β-cells should be included in the regenerative strategies to treat diabetes. Likewise, the capacity of adaptation of newly generated β-cells and the role cell heterogeneity may play in coping mechanisms that respond to different physiological and pathological metabolic challenges *in vivo* is also an open question. Experiments that resolve these matters would highlight pivotal pitfalls in β-cell regeneration approaches aimed at restoring integrated islet function.

## Author Contributions

EB-T, DO, and PH wrote the mini-review. All authors contributed to the article and approved the submitted version.

## Funding

DO was funded by the Hjelt Foundation and PH by the Swiss National Science Foundation (310030_L92496), the Fondation Aclon and the European Research Council (884449-Merlin).

## Conflict of Interest

The authors declare that the research was conducted in the absence of any commercial or financial relationships that could be construed as a potential conflict of interest.
